# Carcinogenesis: alterations in reciprocal interactions of normal functional structure of biologic systems

**DOI:** 10.1186/s13637-015-0030-9

**Published:** 2015-11-18

**Authors:** Garri Davydyan

**Affiliations:** Ottawa Hospital, Ottawa, Ontario K1H 8L6 Canada

**Keywords:** Complex systems, Reciprocal interactions, Negative feedback, Positive feedback, Split quaternions, Carcinogenesis

## Abstract

The evolution of biologic systems (BS) includes functional mechanisms that in some conditions may lead to the development of cancer. Using mathematical group theory and matrix analysis, previously, it was shown that normally functioning BS are steady functional structures regulated by three basis regulatory components: reciprocal links (RL), negative feedback (NFB) and positive feedback (PFB). Together, they form an integrative unit maintaining system’s autonomy and functional stability. It is proposed that phylogenetic development of different species is implemented by the splitting of “rudimentary” characters into two relatively independent functional parts that become encoded in chromosomes. The functional correlate of splitting mechanisms is RL. Inversion of phylogenetic mechanisms during ontogenetic development leads cell differentiation until cells reach mature states. Deterioration of reciprocal structure in the genome during ontogenesis gives rise of pathological conditions characterized by unsteadiness of the system. Uncontrollable cell proliferation and invasive cell growth are the leading features of the functional outcomes of malfunctioning systems. The regulatory element responsible for these changes is RL. In matrix language, pathological regulation is represented by matrices having positive values of diagonal elements (*TrA* > 0) and also positive values of matrix determinant (*detA* > 0). Regulatory structures of that kind can be obtained if the negative entry of the matrix corresponding to RL is replaced with the positive one. To describe not only normal but also pathological states of BS, a unit matrix should be added to the basis matrices representing RL, NFB and PFB. A mathematical structure corresponding to the set of these four basis functional patterns (matrices) is a split quaternion (coquaternion). The structure and specific role of basis elements comprising four-dimensional linear space of split quaternions help to understand what changes in mechanism of cell differentiation may lead to cancer development.

## Introduction

The regulatory structure of normal biologic systems (BS) predisposes the development of functional states mimicking cancer growth. Existing data suggest that cancer cells develop from the stem cells having malignant potential-cancer stem cells (CSC) [1–5]. It was also shown that dysplastic cell disorders (DCD) have common features with cancer development. DCD are even considered as being involved in the staging of carcinogenesis [6–9].

Despite some distinctive properties of carcinogenesis, common features with dysplastic disorders suggest that development of cancer is not a totally self-organizing and autonomous process. The functional structure of cell interactions in normally functioning biologic systems seems to reflect mechanisms of cell differentiation, and the appearance of cancer cells may be caused by some deviations in mechanisms decoding specific patterns of genetic information. Two distinct features of cancer—invasive growth and the ability to metastasize—indicate a specific way of involvement of cancer cells in surrounding tissue or, more precisely, in the functional structure of the normal system. Each biological cell involved in normal system behaviour contributes to maintain the functional and morphological architecture of comprising tissue. When cancer cells develop, they are not involved in the system relationships with normal cells. Containing some functional features of normal cells, cancer cells are only partially restricted (functionally and morphologically) by the surrounding tissue. It makes them not entirely recognizable and controllable by the immune system. In general, immunologic surveillance is not capable of fully controlling and eliminating cancer cells from a population of normal cells. It seems that the functional structure of cancer cells somehow is “embedded” in the structure of normal cell behaviour. To understand the place of carcinogenesis in normal biologic organization, we need to follow two steps:

1. Major functional determinants of phylogenetic extraction and ontogenetic release of biological information should be determined.
2. Of these mechanisms (determinants) responsible for normal regulatory structure of BS, affected components leading to cancer development should be found out.

## Basis functional elements of a biologic system

In a special sense, a BS is considered a collection (constellation) of biologic substances (biologic matter) specifically organized into separable units such as biologic cells, tissues, organs and organisms.

Each of these or other observable morphological or molecular structures obey principles of system organization, so that systems (subsystems) operate as autonomous functional units. Autonomy should be understood as an autoregulatory process of maintaining morphological and functional features to obtain specific system outcome [10–12]. A key factor for the stable outcome is a steadiness of the system’s internal structure. When the system’s structure is steady it means that each of the basis components remains topologically equivalent to the phase portraits representing negative feedback (NFB), positive feedback (PFB) and reciprocal links (RL) in dynamic models [12].

The role of the surrounding environment is that it determines specific inner and outer functional characteristics of the system. Collaboration with the neighbourhood is a key factor for a system to develop specific and steady functional organization. For example, an ocean makes its habitants functionally and morphologically steady in the water, because they developed a special system to extract oxygen. Their functional systems (organs), extremities and body are perfectly adapted to live and survive in this environment. This is also understandable from the general point of view, because any functional system being involved in the functional structure of a higher level can be considered as part of it, and at the same time, the system itself consists of some parts (subsystems) of a lower level. Thus, each arbitrarily chosen element has a neighbourhood as a functional environment, which is linked to this element through the system’s internal regulatory structure. Any artificial element inserted in the system’s internal structure will be considered as “a foreign body” and eliminated. A unique strategy of systemogenesis is seen in perfectly matching parts making different biological systems steady.

Different types of BS have a common feature, which is a uniformly organized internal functional structure. It was proposed that an internal functional structure comprises a space of regulatory elements, presented as combinations of basis functional patterns [12]. In the given environment, functional elements of this space acquire specific functional and morphological “phenotype”. Regulatory elements of the system act on the space of biologic variables, module *L* ≃ *R* ^*n*^. Variables are observables having a biologic nature, which can be measured experimentally.

Based on clinical observations and numerous data regarding functional mechanisms of biologic systems (BS) [13–16], it was proposed that three regulatory patterns, NFB, PFB and RL, form a universal functional basis of the regulatory structure of BS [12, 17]. This conception was supported by a matrix representation of the basis functional elements: $$ {S}_0=\left(\begin{array}{cc}\hfill \hfill & \hfill 1\hfill \\ {}\hfill -1\hfill & \hfill \hfill \end{array}\right) $$ for NFB, $$ {S}_1=\left(\begin{array}{cc}\hfill 1\hfill & \hfill \hfill \\ {}\hfill \hfill & \hfill -1\hfill \end{array}\right) $$ for RL and $$ {S}_2=\left(\begin{array}{cc}\hfill \hfill & \hfill 1\hfill \\ {}\hfill 1\hfill & \hfill \hfill \end{array}\right) $$ represents PFB. The set of the same matrices $$ \left\{\left(\begin{array}{cc}\hfill \hfill & \hfill 1\hfill \\ {}\hfill -1\hfill & \hfill \hfill \end{array}\right),\left(\begin{array}{cc}\hfill 1\hfill & \hfill \hfill \\ {}\hfill \hfill & \hfill -1\hfill \end{array}\right),\left(\begin{array}{cc}\hfill \hfill & \hfill 1\hfill \\ {}\hfill 1\hfill & \hfill \hfill \end{array}\right)\right\} $$ also represents a basis of the Lie algebra *g* ∈ *sl (2,R)* of the special linear group *SL(2,R)*. The basis elements of *g* span a three-dimensional space *R* ^3^ of regulatory patterns that allows demonstrating metric and integrative properties of functional elements of BS [12, 18]. Because of the structural properties of *g*, the geometry of the space of regulatory elements of BS has indefinite metric with the signature (−,+,+) endowing BS with a highly flexible regulatory structure [12].

## Transformation of basis regulatory elements leading to unsteadiness of the system

What is the physiological significance of the basis functional elements? Basis regulatory patterns *S*_0_, *S*_1_, *S*_2_ and their linear combinations with real coefficients span the *space* of functional elements *Λ* (or matrices) *S* = {∑*a* ^*i*^ *S*_*i*_ ∈ *Λ*, *a* ^*i*^ ∈ *R*, *i* = 0, 1, 2} transforming the states of the system. The property of matrices to form linear combinations of the elements of *Λ* reflects an ability of the basis elements as well as other elements of *Λ* to form *integrative* regulatory units. A functional structure of three basis regulatory elements allows the system to correct current functional states so that they could fluctuate strictly within normal functional limits. This is because the matrices of the space of transformations (system’s regulatory structure) are traceless (the sum of diagonal elements is zero), and similarity transformations (as well as a Lie bracket) of any of these matrices will give again a traceless matrix.

*S*_1_ is an operator (matrix) corresponding to reciprocal interactions [12]. It operates according to its matrix properties: applied to a vector [*x*, *y*]^*T*^ characterizing some state of the system; *S*_1_ leaves the first component in the same subspace, while it transforms the direction of the second component to the opposite: *S*_1_ ⋅ [*x*, *y*]^*T*^ = [*x*, − *y*]^*T*^. Its distinctive feature is to divide a space of variables *L* ≃ *R* ^2^ on two one-dimensional invariant subspaces *L*_1_ and *L*_2_. Each of the reciprocal counterparts belongs to the corresponding invariant subspace, where a variable may change its value independently on another one. Thus, this operator splits the whole space (module *L*) on two separable autonomous subspaces, and it transforms components of vectors separately as well. For example, *v* = [1, 0]^*T*^ is an eigenvector of *S*_1_ (it belongs to one-dimensional subspace *L*_1_, which is the *X*-axis). *S*_1_ applied to *v* results in *S*_1_ ⋅ *v* = [1, 0]^*T*^, meaning *S*_1_ did not change the length and direction of *v*, thus leaving *v* unchanged and in the same subspace. In general, *S*_1_ being applied to vectors of invariant subspaces, which are *X*- and *Y*-axes, will leave them in the same subspaces.

Thus, the substantial feature of the “reciprocal” operator *S*_1_ (operator of reciprocal links (RL)) is that it is reducible to one-dimensional components. Each one-dimensional operator acts on its invariant subspace. The functional importance of this operator is based on its ability to split the “homogeneous” functional outcome of the system into two autonomous parts.

**Fig. 1:**
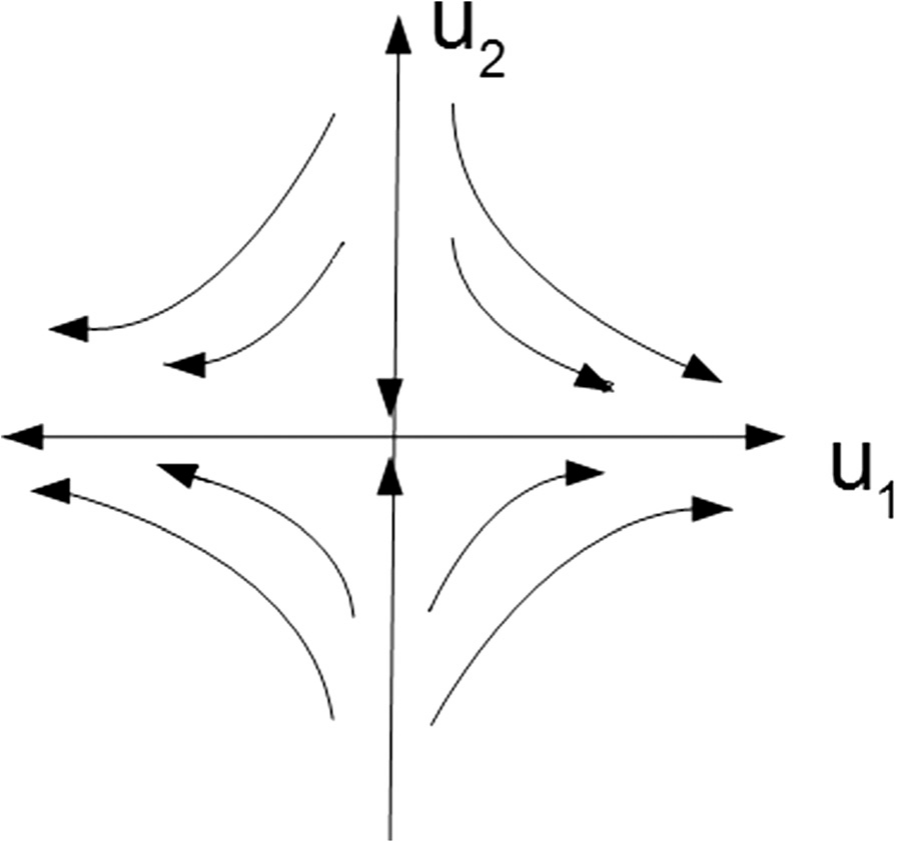
Phase trajectories corresponding to the system $$ \frac{du}{dt}={S}_1u.\ {S}_1=\left(\begin{array}{cc}\hfill 1\hfill & \hfill \hfill \\ {}\hfill \hfill & \hfill -1\hfill \end{array}\right),u=\left({u}_1,{u}_2\right) $$

If *S*_1_ is considered as an operator of ordinary differential equations (ODE) of two variables *x* and *y*, it will give a dynamical image consistent with the saddle. Moving along the saddle trajectory will change the values of variables, leaving the product *x* ⋅ *y* unchanged (*xy* = const) (Fig. 1), [12, 20].

**Fig. 2:**
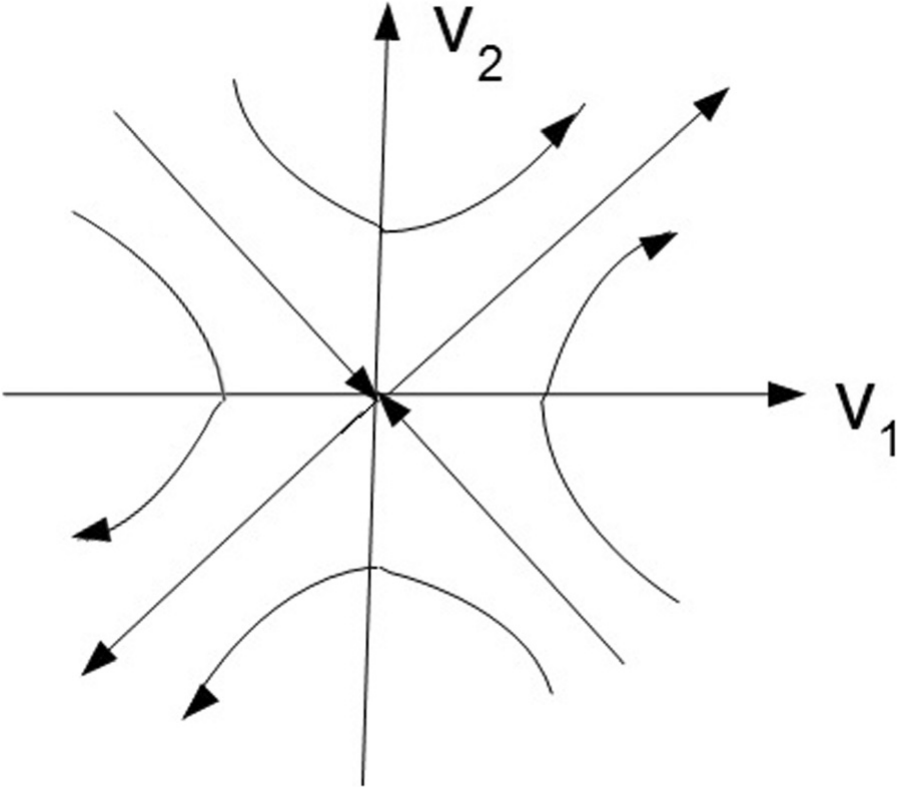
Phase trajectories corresponding to the system $$ \frac{dv}{dt}={S}_2v.{S}_2=\left(\begin{array}{cc}\hfill \hfill & \hfill 1\hfill \\ {}\hfill 1\hfill & \hfill \hfill \end{array}\right),v=\left({v}_1,{v}_2\right) $$

One-dimensional invariant subspaces are the four pieces of coordinate axes and zero point, meaning that evolution of the system, if started at any point of the coordinate axis, will evolve along this axis, i.e., states belonging to an invariant one-dimensional subspace (coordinate axis) will remain in and not leave this space. The same trajectories but rotated counterclockwise on 45° around the zero point correspond to the matrix of PFB, $$ {S}_2=\left(\begin{array}{cc}\hfill \hfill & \hfill 1\hfill \\ {}\hfill 1\hfill & \hfill \hfill \end{array}\right) $$ (Fig. 2).

Each phase curve of *S*_1_ and *S*_2_ matrices lies on the surface of a constant energy level [19]. This means that if a whole system consists of two parts, and one of them produces energy and another consumes it, then during the system’s evolution the total amount will be “distributed” between subsystems remaining unchanged. Thus, the systems, described by these matrices, do not depend on additional sources of energy and can be considered stable, because the regulatory machinery works to distribute, not dissipate, an energy keeping its total amount at the same level.

**Fig. 3:**
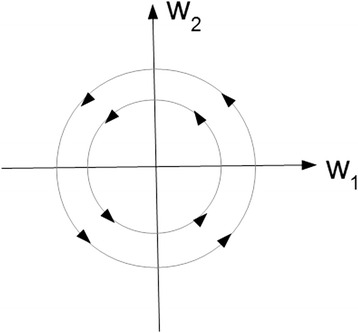
Phase trajectories corresponding to the system $$ \frac{dw}{dt}={S}_0w.{S}_0=\left(\begin{array}{cc}\hfill \hfill & \hfill 1\hfill \\ {}\hfill -1\hfill & \hfill \hfill \end{array}\right),w=\left({w}_1,{w}_2\right) $$

The matrix corresponding to the NFB, $$ {S}_0=\left(\begin{array}{cc}\hfill \hfill & \hfill 1\hfill \\ {}\hfill -1\hfill & \hfill \hfill \end{array}\right) $$, shows negative feedback regulatory patterns by concentric circles (Fig. 3). Each of the circles also lies on the surface of the same energy level likewise phase trajectories of *S*_1_ and *S*_2_ [19]. Thus, the regulatory structure of BS described by the elements of the space of transformations *Λ* spanned by {*S*_0_, *S*_1_, *S*_2_} substantially cannot lead to unsteady states. Even for matrices in the general view $$ S=\left\{\left(\begin{array}{cc}\hfill a\hfill & \hfill b\hfill \\ {}\hfill c\hfill & \hfill d\hfill \end{array}\right)\in \varLambda, a+d=0\right\} $$, similarity transformations will again give traceless matrices.

Classical steady or unsteady states of the system can arise if the corresponding “reciprocal” matrix (operator) $$ {S}_1=\left(\begin{array}{cc}\hfill 1\hfill & \hfill \hfill \\ {}\hfill \hfill & \hfill -1\hfill \end{array}\right) $$ is transformed to the matrix whose trace is not zero $$ {S_1}^{\prime }=\left(\begin{array}{cc}\hfill a\hfill & \hfill \hfill \\ {}\hfill \hfill & \hfill b\hfill \end{array}\right) $$, for example, *a,b* > 0 or *a,b* < 0, *a* + *b* ≠ 0. ${S}_1^{\prime }$ can be normalized to have the view $$ \pm E=\pm \left(\begin{array}{cc}\hfill 1\hfill & \hfill \hfill \\ {}\hfill \hfill & \hfill 1\hfill \end{array}\right) $$. Integrative regulatory unit of the system with the added regulatory element *aE* will correspond to the linear combination of the matrices *Q* = *aE* + *bS*_1_ + *cS*_2_ + *dS*_0_ with real coefficients. Because *TrQ* ≠ 0, the Jordan normal form may have any of four possible types of matrices [19, 20] including the ones which correspond to steady or unsteady phase trajectories.

**Fig. 4:**
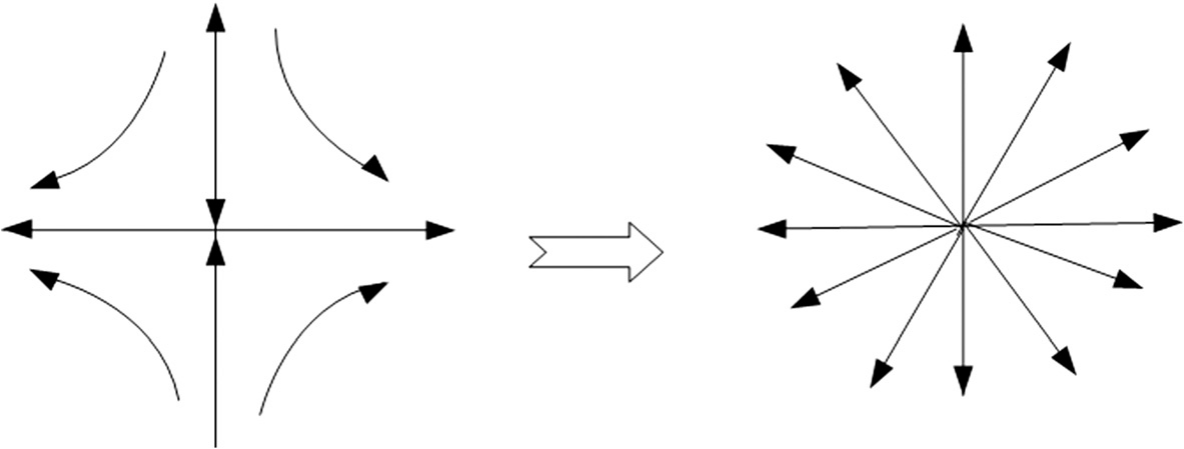
Bifurcation in the system’s behaviour is caused by changes in the system’s parameters. A saddle transforms to the unsteady node. Two systems are not topologically equivalent—they cannot be transformed to one another by smooth transformations

If *a* > 0 and *b* > 0, then $det{S}_1^{\prime }>0$ and ${S}_1\to {S}_1^{\prime }$ is not a smooth transformation due to different signs of determinants of *S*_1_ and ${S}_1^{\prime }$. Thus, the deterioration of reciprocal interactions resulting in the transformation of a saddle into an unsteady node occurs through the bifurcation^1^ of the phase trajectories (Fig. 4).

**Fig. 5:**
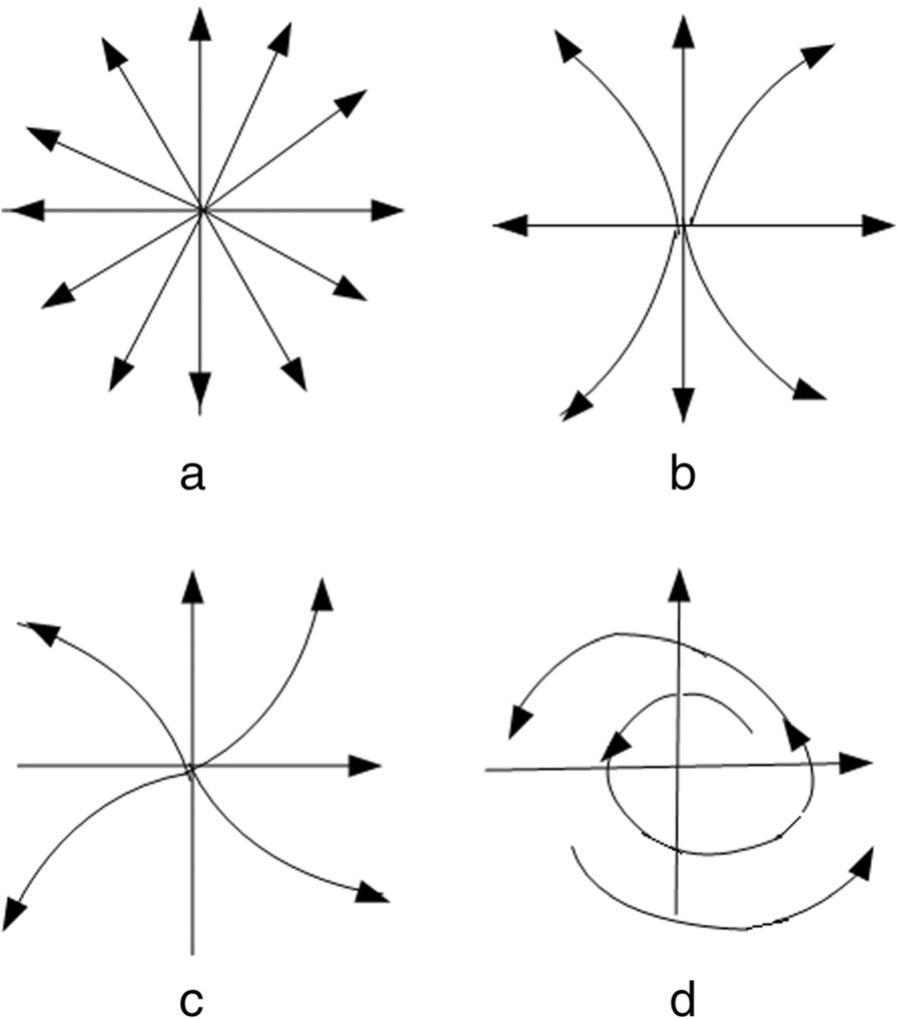
Unsteady systems shown by phase trajectories of ODE $\frac{dx}{dt}= Ax,TrA>0, detA>0$: **a** unsteady stellar node corresponding to matrix $$ A=\left(\begin{array}{cc}\hfill p\hfill & \hfill \hfill \\ {}\hfill \hfill & \hfill p\hfill \end{array}\right) $$; **b** unsteady node corresponding to $$ A=\left(\begin{array}{cc}\hfill m\hfill & \hfill \hfill \\ {}\hfill \hfill & \hfill n\hfill \end{array}\right) $$; **c** improper node corresponding to $$ A=\left(\begin{array}{cc}\hfill m\hfill & \hfill 1\hfill \\ {}\hfill \hfill & \hfill m\hfill \end{array}\right) $$; **d** unsteady focus corresponding to $$ A=\left(\begin{array}{cc}\hfill a\hfill & \hfill b\hfill \\ {}\hfill -b\hfill & \hfill a\hfill \end{array}\right) $$

Therefore, this type of change in the reciprocal regulatory structure leads to unsteady states. Because of the alteration of regulatory mechanisms leading to the transformation of “reciprocal” matrix *S*_1_ into a diagonal matrix with positive eigenvalues ${S}_1^{\prime }$, a new operator ${S}_1^{\prime }$ has become involved in the regulatory process, and a system (subsystem) acquires a *tendency* towards unsteadiness. The portion of the regulatory process related to the altered (2 × 2) block in an *n*-dimensional operator representing the whole system will lead to some pathological states of the corresponding subsystem. If NFB and PFB components remain intact, behaviour of the system will evolve according to the unstable (stellar) node (Fig. 5a, b).

**Fig. 6:**
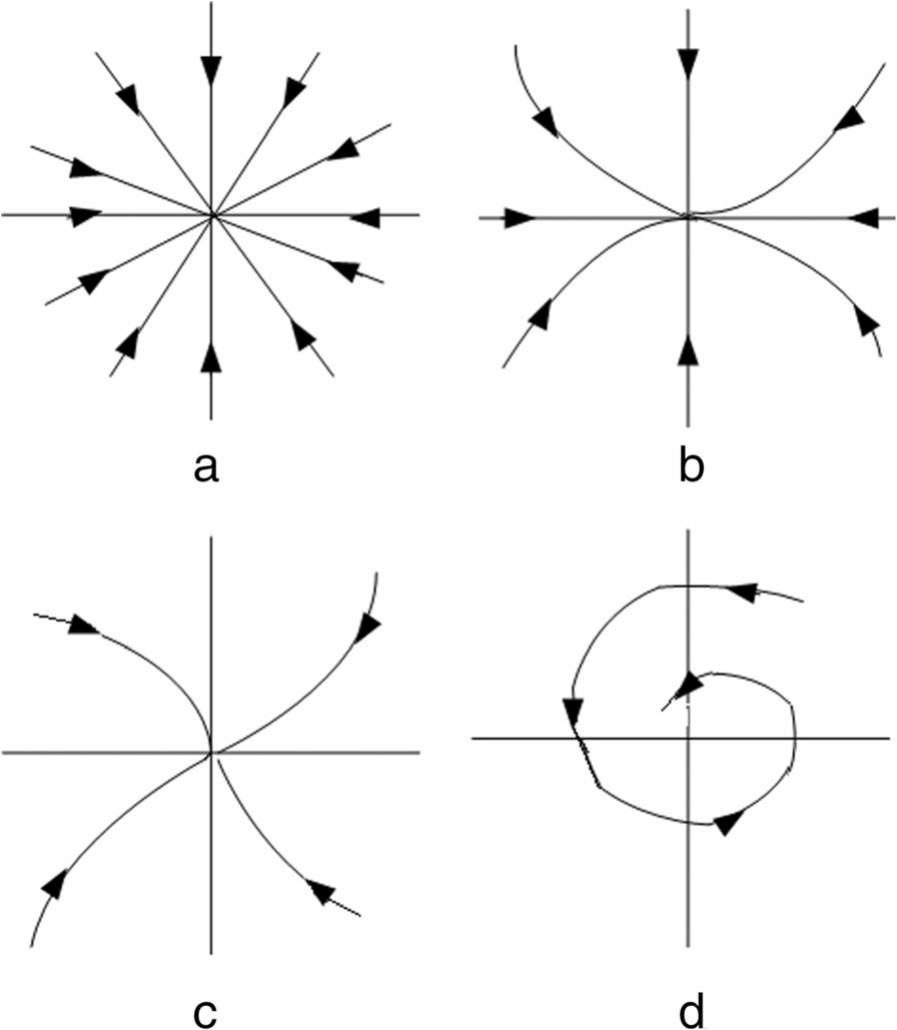
Steady systems shown by phase trajectories of ODE $\frac{dx}{dt}= Ax,TrA<0, detA<0$: **a** steady stellar node corresponding to matrix $$ A=\left(\begin{array}{cc}\hfill -p\hfill & \hfill \hfill \\ {}\hfill \hfill & \hfill -p\hfill \end{array}\right) $$; **b** steady node corresponding to $$ A=\left(\begin{array}{cc}\hfill -m\hfill & \hfill \hfill \\ {}\hfill \hfill & \hfill -n\hfill \end{array}\right) $$; **c** improper node corresponding to $$ A=\left(\begin{array}{cc}\hfill -m\hfill & \hfill 1\hfill \\ {}\hfill \hfill & \hfill -m\hfill \end{array}\right) $$; **d** steady focus corresponding to $$ A=\left(\begin{array}{cc}\hfill -a\hfill & \hfill b\hfill \\ {}\hfill -b\hfill & \hfill -a\hfill \end{array}\right) $$

If *a* < 0 and *b* < 0, then *detS*_1_^′ ′^ > 0, *TrS*_1_^′ ′^ < 0, and the subsystem will evolve towards steady state (Fig. 6).

Therefore, to describe the steadiness or unsteadiness of a system’s behaviour as it is used in classical dynamical modelling, a unit matrix $$ \pm E=\pm \left(\begin{array}{cc}\hfill 1\hfill & \hfill \hfill \\ {}\hfill \hfill & \hfill 1\hfill \end{array}\right) $$ to the set of the basis matrices {*S*_0_, *S*_1_, *S*_2_} of Lie algebra *g* ∈ *sl(2,R)* should be added. In fact, adding a unit matrix will allow describing pathological conditions of BS, because the substantial feature of pathological states is the unsteadiness of the system, leading to the deterioration of its functional and morphological structure. Including a unit matrix also results in a new mathematical structure, split quaternions (coquaternions), discussed further in the text.

## Realization of altered matrix structure in some pathological conditions

In the conditions when BS reacts by inflammation evoked, for example, by the invasion of pathogenic microorganisms, deteriorated cells and tissues may already have mature features. Right after birth, a biological system becomes autonomous and independent. After the birth, the system contains all anatomic and functional elements needed to survive and develop in the surrounding environment. All that is needed is to obtain better adaptation to the existing environment through maturation. Functional and morphological maturation mean an increase of the system’s functional productivity using hidden metabolic reserves for cells, tissues and organs. These reserves are the following: an increase of production of hormones, proliferation of the existing cells and formation of new neural regulatory links. For example, physical training may increase muscle strength, due to muscle hypertrophy and mitochondrial hyperplasia, not affecting the structure of myofibrils and without substantial changes in muscle metabolism. This is an example of how the system can improve its functionality without qualitative changes of the inner structure. Some people can be trained so that they can swim very fast like a fish, but they do not develop anatomical and functional features inherent to fish.

An inflammation is aimed to replace deteriorated tissue by regeneration through the asymmetric division of stem (cambial) cells having “minimal” immature potency. The level of differentiation of cambial cells seems to correspond to the level of deterioration caused by the severity of invasion and inflammatory reaction on it. The internal regulatory structure of BS, which normally leads metabolism, cell interactions and apoptosis, is not affected by an inflammation. Regeneration of tissues and organs after microbial invasion or trauma is a result of an adequate system’s activity materialized in excessive output (granulation tissue). In cases of inflammatory reactions, a system increases its functional turnover initiating new biochemical reactions and activating additional sources for regenerative processes. Asymmetric and symmetric cell divisions with the involvement of stem cells (SC) are the main source for reparation of affected tissue. Genetic mechanisms of tissue reparation including RL, PFB and NFB functional elements remain intact. Tissue reparation is a part of the cell renewal process. The reaction of the system to inflammation does not lead to self-destruction. Hyperproliferative activity seen in inflammatory reactions indicates the compensatory attempts to restore normal tissue. Newly formed tissue does not have dysplastic features.

Systemic diseases differ from the banal inflammatory reactions caused by bacterial invasion or traumatic injuries in that they are developed as autoimmune reactions on antigens produced by the system itself. Unlike inflammatory reactions on invasion of pathogenic microbial agents or mechanical injuries, autoimmune disease usually takes a chronic course because of permanent attacks of antigen-antibody complexes on renewed tissue. An antigen-antibody complex is an intrinsic permanently acting additional deteriorating pathophysiological component, which interferes with the process of tissue renewal, thus preventing normal regeneration. In cases of autoimmune inflammation, there are two components of pathophysiologic mechanisms: formation of immune complexes affecting specific tissue and the process of regeneration attempting to restore deteriorated tissue. Each of these processes does not affect functional mechanisms responsible for the formation of normal tissue or organogenesis. Indeed, the formation of autoimmune complexes does not originate from the cell’s differentiation mediated by reciprocal regulatory mechanisms or from tissue reparation, which uses an unaltered symmetric and asymmetric division of stem cells of different origin to restore epithelial cells, connective tissue and possibly other components from which affected tissue is formed.

**Fig. 7:**
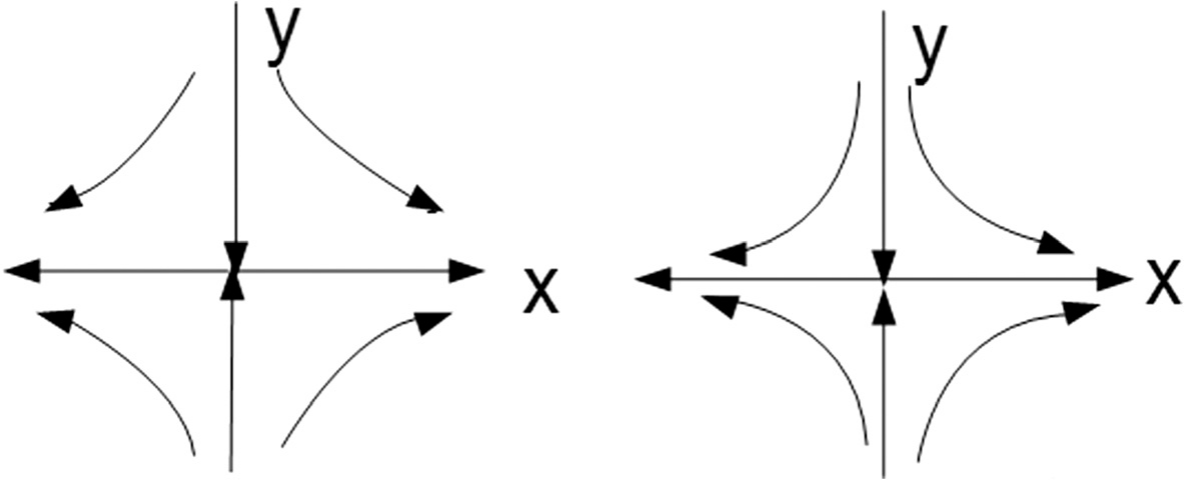
Two saddles corresponding to the matrices $$ A=\left(\begin{array}{cc}\hfill 1\hfill & \hfill \hfill \\ {}\hfill \hfill & \hfill -1\hfill \end{array}\right) $$ (*left image*) and $$ A=\left(\begin{array}{cc}\hfill n\hfill & \hfill \hfill \\ {}\hfill \hfill & \hfill -1\hfill \end{array}\right),n>1 $$ (*right image*). (See explanations in the text)

Benign tumours in order to develop must have altered reciprocal mechanisms. They still are involved in a systemic relationship with other tissue components, because benign tumours do not have invasive features. Surrounding morphological structures remain intact, except they are incapable of suppressing the proliferative activity of benign neoplasms. The range of suppression capabilities depends on the type of tumour. For example, desmoid fibromas have practically uncontrollable growth. Although not having invasive potential, these tumours clinically behave like malignant ones, because of extremely fast (could be few weeks) growth that within weeks may cause the deadly compression of organs and blood vessels. Some other benign neoplasms, for example, naevuses, lipomas and fibromas, usually slow down their proliferative activities remaining unchanged for years. Perverted reciprocal regulatory structures of benign neoplasms, yet malfunctioning, prevent bypassing sources of energy from surrounding tissues. In matrix language, this type of regulation can be expressed by matrices $$ A=\left(\begin{array}{cc}\hfill n\hfill & \hfill \hfill \\ {}\hfill \hfill & \hfill -1\hfill \end{array}\right) $$, *n* > 1, *TrA* > 0, and *detA* < 0 (Fig. 7). Phase portrait of the system still corresponds to the saddle, but the character of the curves demonstrates a rapid increase in the values corresponding to the *x* variable and relatively slowly diminishing values of the *y* variable. It could be interpreted as an unproportional activation of one subsystem (proliferation) that is not entirely counteracted by another (proliferation suppressor factor).

Malignant tumours have some prominent features that make them qualitatively different in the behavioural patterns compared to the previously described pathological conditions. Carcinomas are characterized by invasive growth and ability to metastasize. Malignant cells, remaining functionally and morphologically undifferentiated structures, represent a wide spectrum of cells with different malignant potentials from well- to moderately- and up to the low-differentiated types. Low-differentiated cells have the highest malignant potential. The behaviour of cancer cells does not obey functional restrictions from the neighbouring structures. Surrounding tissues as well as other organs serve cancer cells as a source of energy, which together with the inner structure of cancer cells is the only limitation factor for efficient cancer growth. Cancer as a functional and morphological derivative of the normal system has a highly unsteady structure. Its morphological and functional unsteadiness manifest in the destruction of not only surrounding and distant tissues but also its own cells that eventually lead to the death of the whole system (organism). In spite of an avalanche-like “deteriorating” PFB regulatory mechanism, some formal characteristics of PFB contradicts the fact it may have destructive behaviour. First of all, PFB is one of the *basis* regulatory elements of the biologic system’s regulatory structure. Its representation in ODE shows that PFB is a conservative system, i.e. its phase trajectories (saddle) lie on the surface of the same energy level, so that evolution of the system’s states does not need additional sources of energy.

*S*_1_ and *S*_2_ have *detS*_1_ < 0 and *detS*_2_ < 0, so both matrices can be transformed to one another by continuous transformations. No bifurcations of phase portrait corresponding to the “catastrophic” behaviour are needed.

It is important that mutual transformations change the basis in which matrices are presented. The physiological meaning of basis transformations is that it allows observing primary elements and forming from them systems by using different angles of observation [12]. It also allows the new systems to be composed with separable characteristics viewed, for example, as tissues and organs. The matrix representing NFB is the only one having a positive determinant that explains matrix properties to have continuous paths connecting it to the matrices with a positive determinant, including the +*E* (unit) matrix. Because it is also an orthogonal matrix, applied (multiplied) from the left to *S*_1_ and(or) *S*_2_, it rotates phase trajectories through the axis orthogonal to the plane of the phase images so that they can coincide. One of the physiological meanings of *S*_0_ is to represent complex systems as a composition of simpler parts. By analogy, it can be seen when *S*_0_ transforms *S*_1_ to *S*_2_, *S*_1_ *S*_0_ = *S*_2_. If *S*_1_ is expressed in a standard basis, then the basis vectors [1, 0]^*T*^, [0, 1]^*T*^ are eigenvectors of invariant subspaces which are coordinate axes. After *S*_1_ is transformed to *S*_2_, basis vectors corresponding to the eigenvectors of invariant one-dimensional subspaces of *S*_2_ became [1, 1]^*T*^, [−1, 1]^*T*^. This means that vectors corresponding to the initial physiologic variables are perturbed, so that each of the new variables will contain components of the initial variables. It can be interpreted in a way that depending on combinations of basis elements a system acquires a new representation and has a view where the composition of primary elements is presented in some combinations satisfying functional demands to the newly formed systems. For example, combination of epithelial cells, glandular cells, connective tissues and their derivatives may represent a mucous membrane of a specific organ. If, in turn, the structure of composed elements (tissues and cells) remains unchanged, then following the same procedure the next integrative level will correspond to organs. The diagonal form of *S* gives primary (pure) functional elements through eigenvectors of invariant one- or two-dimensional subspaces. In an *n*-dimensional module (space of physiologic variables), a diagonal form obtained after orthogonal transformations again represents a reciprocal matrix with equal numbers of positive and negative entries. In the space of quadratic forms of *n* variables, it corresponds to the *n*-dimensional hyperboloid oriented along new coordinate axes.

From the physiological point of view, the only bifurcation leading to unsteady systems is when a reciprocal matrix with different signs of diagonal entries “transforms” to a diagonal form having positive entries so that *detS*_1_′ > 0, *TrS*_1_′ > 0 (Fig. 4). No linear combinations of the basis elements *S*_0_, *S*_1_, *S*_2_ can give a matrix with the above properties. Unsteady systems lie apart from the structural properties given by elements of Lie algebra *g* ∈ *sl(2,R)*. Matrices (*A*) describing unsteady systems must satisfy two conditions: *detA* > 0, *TrA* > 0. This condition corresponds to the diagonal matrix with positive entries, and it describes dynamic systems viewed as unsteady nodes.

If an integration of RL, NFB and PFB provides functional regulation within the strict frames of normal development of BS, to describe all possible functional states of the system including pathological conditions, an additional functional element(s) should be included in the regulatory structure. Formally, pathological states may arise as a result of the sequential applications of normal functional (regulatory) elements to a current state of the system, which in matrix language means matrix multiplication.

Multiplication is not a group operation of *g*. Multiplication of matrices of *g* ∈ *sl(2,R)* gives elements not belonging to *g*. To obtain a functional structure closed under multiplication, a unit matrix $$ 1=\left(\begin{array}{cc}\hfill 1\hfill & \hfill \hfill \\ {}\hfill \hfill & \hfill 1\hfill \end{array}\right) $$ to the set of the basis elements {*S*_0_, *S*_1_, *S*_2_} has to be added.

An appearance of a unit matrix as the basis component in the formal regulatory structure of BS brings new functional actions that may change the structure of reciprocal interactions. These changes correspond to the deterioration of “negative”, inhibitory links and replacing them with positive, system-activating stimuli. Thus, inhibiting a reciprocal component transforms it to an excitatory one that substantially changes the subsystem’s behaviour leading to unsteadiness. Deterioration of regulatory mechanisms following this scenario is proposed to be a leading cause of malignant transformations. Other changes in reciprocal interaction reflected in the *S*_1_ matrix structure (*TrS*_1_ > 0, *detS*_1_ < 0) (Fig. 7) will correspond to the “milder” pathological conditions (for example, benign tumours) discussed above.

## A coquaternion ring as a space for modelling pathological states of biologic systems

The set $$ G=\left\{\pm \left(\begin{array}{cc}\hfill 1\hfill & \hfill \hfill \\ {}\hfill \hfill & \hfill 1\hfill \end{array}\right),\pm \left(\begin{array}{cc}\hfill 1\hfill & \hfill \hfill \\ {}\hfill \hfill & \hfill -1\hfill \end{array}\right),\pm \left(\begin{array}{cc}\hfill \hfill & \hfill 1\hfill \\ {}\hfill 1\hfill & \hfill \hfill \end{array}\right),\pm \left(\begin{array}{cc}\hfill \hfill & \hfill 1\hfill \\ {}\hfill -1\hfill & \hfill \hfill \end{array}\right)\right\} $$ is a multiplicative group, where the last three components represent the basis elements of Lie algebra *g* ∈ *sl(2,R)*. *G* is also a basis of an algebra of 2 × 2 matrices over *R*. If elements of *G* are denoted as $$ 1=\left(\begin{array}{cc}\hfill 1\hfill & \hfill \hfill \\ {}\hfill \hfill & \hfill 1\hfill \end{array}\right),\kern0.5em i=\left(\begin{array}{cc}\hfill \hfill & \hfill 1\hfill \\ {}\hfill -1\hfill & \hfill \hfill \end{array}\right),\kern0.5em j=\left(\begin{array}{cc}\hfill 1\hfill & \hfill \hfill \\ {}\hfill \hfill & \hfill -1\hfill \end{array}\right),\kern0.5em k=\left(\begin{array}{cc}\hfill \hfill & \hfill 1\hfill \\ {}\hfill 1\hfill & \hfill \hfill \end{array}\right) $$, the set {1,*i*,*j*,*k*} will represent a basis of a coquaternion (split quaternion) algebra over *R* introduced by James Cockle in 1849 [21]. Considered as an orthonormal basis, {1,*i*,*j*,*k*} spans *R* ^4^ of coquaternions *q* = *a* ^0^ + *a* ^1^ *i* + *a* ^2^ *j* + *a* ^3^ *k*, where *a* ^*i*^ are real numbers.

Using elements of the algebra of split quaternions allows describing a behaviour of linear (or linearized) systems^2^ where matrices of the group are used in the second-order ODE. The behaviour of these systems is well known and classified [19]. Four possible classes of second-order ODE can be obtained after the corresponding matrices in the general view are transformed to the canonical, Jordan, form using similarity transformations. Unlike the conditions when matrices used in ODE belong to *R* ^3^ spanned by basis elements of the Lie algebra *g* ∈ *sl(2,R)*, states arising due to the unit matrix ±*E* of the group *G* allow describing classical steady and unsteady behaviour of dynamical systems. Phase portraits derived from unit matrix represent steady $$ \left(A=\left(\begin{array}{cc}\hfill -1\hfill & \hfill \hfill \\ {}\hfill \hfill & \hfill -1\hfill \end{array}\right)\right) $$ and unsteady $$ \left(A=\left(\begin{array}{cc}\hfill 1\hfill & \hfill \hfill \\ {}\hfill \hfill & \hfill 1\hfill \end{array}\right)\right) $$ nodes (Figs. 5a and 6a). Phase trajectories related to steady and unsteady nodes should be considered as *tendencies* in a system’s behaviour leading in both situations to the deterioration of BS. In the case of unsteady nodes, it is obvious, because increasing demands of the system are not supported by available sources of energy. In cases of steady nodes, equilibrium states could be acceptable options, if diminishing or low functionality is a final goal of BS. It could be considered as a normal state in a short time interval for some extreme conditions but not as a normal long-term strategy in the system’s behaviour.

The classic description of the steadiness of second-order linear systems is given by matrices from *q* as operators of ODE, for example, $\frac{du}{dt}=Au,\kern0.5em A\in q$, whose trace is *TrA* > 0 (unsteady systems) or *TrA* < 0 (steady systems). These matrices transformed to the Jordan form have the view $$ \begin{array}{l}{J}_1=\left(\begin{array}{cc}\hfill {\lambda}_0\hfill & \hfill \hfill \\ {}\hfill \hfill & \hfill {\lambda}_0\hfill \end{array}\right),\kern0.5em {\lambda}_0>0\kern0.75em \mathrm{or}\kern0.5em {\lambda}_0<0;\kern0.5em {J}_2=\left(\begin{array}{cc}\hfill {\lambda}_0\hfill & \hfill \hfill \\ {}\hfill \hfill & \hfill {\lambda}_1\hfill \end{array}\right),\kern0.5em {\lambda}_{0,1}>0\kern0.75em \mathrm{or}\kern0.5em {\lambda}_{0,1}<0,\kern0.5em {\lambda}_0\ne {\lambda}_1;\\ {}{J}_3=\left(\begin{array}{cc}\hfill {\lambda}_0\hfill & \hfill 1\hfill \\ {}\hfill \hfill & \hfill {\lambda}_0\hfill \end{array}\right),\kern0.5em {\lambda}_0>0;\kern0.5em {J}_4=\left(\begin{array}{cc}\hfill \alpha \hfill & \hfill -\beta \hfill \\ {}\hfill \beta \hfill & \hfill \alpha \hfill \end{array}\right)\end{array} $$, *a* and *β* are real numbers (Figs. 5 and 6) [20].

## The strategy of phylogenesis is to obtain one-dimensional invariant subspaces

It is logical to assume that cornerstones of phylogenetic mechanisms of encoding of biologic information are reflected in the internal functional structure of BS.

During phylogenetic development, adaptive functional properties are encoded in the genome as steady patterns. It is conventional to consider that every functional feature (a character) is represented by a single gene. In a much shorter process, ontogenesis (or individual development), encoded in genes morphological and functional properties are being decoded, which is finalized in mature biologic objects. This process is mostly implemented through the asymmetric and, additionally, symmetric cell divisions, during which pluripotent (but functionally rudimentary) cells undergo differentiation until they reach competence in realizing specialized functions.

The stages of cell differentiation can be demonstrated through a functional tree, where from the bottom to the top the cells acquire special features simultaneously with the loss of the potency in expressing more common features possessed by their progenitors. Narrowing the spectrum of cell’s qualitative characteristics has its limits in individual development. The final point in cell differentiation is not an absolute, if considered in the scope of a continuum of phylogenetic development. Even if the cell realizes a highly differentiated function, on a more thorough scale, this function would be seen as relatively general, virtually allowing the widening of the spectrum of realized functions for further cell differentiation. This process lies outside of ontogenetic development and characterizes phylogenesis, the long-term adaptation process, resulting in new morphological and functional features related to them.

To understand how functional splitting may occur and what underlies its mechanism, let us consider an example of the development of the cardiovascular system (CVS) from primitive organisms to animals [22]. In primitive unicellular organisms, there is no specialized CVS. Oxygen is transported through the cell’s surface and distributed inside the cell according to electrochemical gradients. This way is optimal for small bodies with restricted functionality. There is no need to develop pumping organs (heart) and even specialized “channels” for blood supply (blood vessels). In complex organisms, blood vessels are distinguished structures aimed to supply oxygen and specialized blood cells throughout the whole body. To make this process efficient, at least two components must be controllable: rheology of the blood and the wholeness of the vessel’s wall. It is seen that during phylogenetic development the undifferentiated amorphous structure for oxygen transport in primitive organisms has transformed in animals into the specialized organ, the vessels (hemocoel baths), and blood (or hemolimph), separated from surrounding tissues by the vessel’s walls. The structureless gel (functional property A) is differentiated to the solid component (vessel’s walls) (B) and liquid substance (the blood with the former elements) (C). Thus, the functional spectrum of the system aimed at the oxygen supply widened from an amorphous gel (A) to the vascular system having at the one pole “firm” vessel’s walls (B) and at another—“liquid” substance, blood (C). Functional splitting during phylogenesis has occurred in two opposite directions—towards forming channels (containers) with propulsive capability of their walls, the vessels, and a liquid substance having much better rheology than a gel in primitive cellulata. Each component has its own regulatory mechanism, integrated with its antipodal part according to the common functional stem from which differentiated structures have been developed. For example, clot formation and clot degradation cascades are integrated actions maintaining together the whole spectrum of conditions responsible for the viscosity of the blood. It is interesting that activation of the clot formation process unconditionally begins in cases of deterioration of a blood vessel’s wall, and it includes extrinsic pathways activating platelets’ adhesive properties aimed to close defects of the vessel’s wall to prevent bleeding. Activation of the extrinsic coagulation pathway also occurred, if the smoothness of the inner surface (intima) of the blood vessels is deteriorated by atherosclerotic patches.

In primitive organisms, respiratory function is realized by the diffusion of oxygen through the cell’s wall and its distribution according to electrochemical gradients. In animals, respiratory function has developed in two directions: to conduct an air flow and increase the surface of oxygen saturation and carbon dioxide elimination. Therefore, functional and morphological splitting occurred through the development of the trachea-bronchial tree including complex mechanisms facilitating and impeding air flow (work of intercostal muscles, mechanisms of bronchial constriction and relaxation, constriction and relaxation of diaphragmatic muscles, etc.) and tremendously large alveolar surface with alveolar-capillary membrane specially developed to alleviate oxygen saturation and CO_2_ elimination from the blood. The velocity of air flow and alveolar-capillary gradient regulatory mechanisms each are under the control of reciprocal regulatory mechanisms. They are integrated as reciprocal elements as well. It should be noted that they are not in antagonistic but rather are complementary interactions. The skin also realizes respiratory function that mimics the one in primitive organisms and confirms splitting of the primary mechanisms on reciprocal parts.

The same process of morphological and corresponding functional splitting into reciprocal components can be traced during the development of digestive, endocrine, urinary tract systems, etc. More accurately, the phylogenetical splitting results in the appearance of not opposite but rather *orthogonal* functional properties: *X* → *X*_1_ × *X*_2_. In cases when one original functional character splits into two more specific ones, states of the system are represented by points on the plane of two variables that give a more complex area of functional presentation compared to one-dimensional domains of monocomponent undifferentiated systems.

In a standard basis, a matrix representing a functional split is a traceless diagonal 2 × 2 matrix of RL $$ {S}_1=\left(\begin{array}{cc}\hfill 1\hfill & \hfill \hfill \\ {}\hfill \hfill & \hfill -1\hfill \end{array}\right) $$. Because of the equivalency of functional parts, splitting should be represented by $$ \pm {S}_1=\pm \left(\begin{array}{cc}\hfill 1\hfill & \hfill \hfill \\ {}\hfill \hfill & \hfill -1\hfill \end{array}\right) $$.

To demonstrate advantages of adaptation due to phylogenetic splitting, consider the system consisting of two subsystems which maintain rheology of the blood: the clot formation biochemical reactions and the clot degradation cascades. Because of reciprocal interactions between these two subsystems, they can be considered as two orthogonal to each other variables, forming a two-dimensional space each point of which is (roughly) a viscosity condition of the blood. Any curve on the plane of the two variables will show the character of interactions between subsystems denoted by these variables. In “pure” conditions, phase images will correspond to the saddle (Fig. 1). Each of the two abovementioned subsystems can be considered as relatively independent systems containing NFB and PFB loops as components of inner regulatory mechanisms [12].

The phylogenetic sense of RL could be understood as a process of splitting an original “homogeneous” or, more accurately, rudimentary functional outcome into differentiated components in order to broaden an area of functional properties of the system, including the system’s output. This process accomplishes by encoding in chromosomes metabolic pathways for synthesizing substances responsible for encoded function. There is a correspondence between group of genes or even one gene and expressed function. Each gene or group of genes expresses some function. The *structure* of environmental impacts on developing BS is captured in genes as a system’s response to the environmental output. Following the logic of reciprocity, each function should have its “counterpart”. It should be understood not like ±relationships but in a more constructive way. The counterparts of realizing functions should be orthogonal to one another, like *X*_1_ and *X*_2_ orthogonality makes it possible to map the objects corresponding to the points on the plane *X*_1_ × *X*_2_. The properties of the objects are expressed as pairs of characteristics taken from *X*_1_ and *X*_2_ spaces. A product of two orthogonal spaces as the spaces of biologic variables forms two-dimensional objects. RL between two variables is expressed by a corresponding matrix (*S*_1_). For *n*-dimensional spaces *X*_1_ × *X*_2_ × … × *X*_*n*_, a matrix of RL is a diagonal scalar matrix with equal numbers of positive and negative entries. For example, a liver—a digestive, detoxifying, protein-synthesizing organ with other multiple important functions—consists of liver cells (hepatocytes), bile ducts (including epithelial cells), blood vessels, connective tissue elements, etc. It can be considered as an *n*-dimensional object, if each type included in the liver tissue or other separable elements is given as a variable determining liver function. Reciprocal parts of blood vessels were discussed above. Bile ducts’ reciprocal components can be considered by analogy with blood vessels as peristaltic tubules conducting specific liquid substances. As complex metabolic machinery, a liver also has reciprocal elements in metabolic pathways regulating anabolic and catabolic components, glycogen synthesis vs glycogenolysis, protein synthesis vs gluconeogenesis, lipolysis vs lipoprotein and cholesterol synthesis, etc.

For instance, bile acid production depends on blood components and nutrients (amino acids, lipids, including cholesterol, glucose, electrolytes, etc.), supplied by the digestive tract via the portal vein. The amount of these functional components being involved in metabolism depends on the consuming needs of other organs as well as on the functional capabilities of liver suppliers providing substrates for the liver function. Therefore, some of the metabolic pathways of the liver are involved in competitive metabolism.

For example, a reduction in bile acid production is accompanied by increase in synthesis of fatty acid and triglycerides in the liver and secretion into blood [23].

Some of the regulatory mechanisms are under control of NFB [24]. It should be noticed that NFB and RL have common features clearly seen in the portions of their phase trajectories. To distinguish them, it is important that the whole paths are being traced, which in NFB should be a closed circle with a simultaneous increase or decrease of activities of both variables alternating with the portions of the curve where activation of one subsystem accompanied by suppression of another as it is seen in reciprocal interactions. Reciprocal interactions are always opposite in direction: an increase in activity of one subsystem accompanied by diminished output of another and vice versa.

Each function realized by a functional system is distributed within the spectrum of its characteristics, where marginal parts are the most distant in their functional properties and the most prominent feature(s) is located in the centre of the area of distribution.

The spectrum of the system’s output determines the system’s functionality provided it is adequate in the existing environment. Changes in the environment, if they are not abrupt and damaging, will cause a system’s adaptation, first by modifying its behaviour, and later by encoding the most adaptive of these behavioural patterns in genes, so that a mature organism (organ, tissue) genetically will be capable to survive.

Phylogenetic development is a process of extracting incoming patterns from the environment and encoding them as reciprocal functions. Thus, splitting of monofunctional (homogeneous) input into two autonomous parts goes simultaneously with the formation of autonomous mechanisms controlling each reciprocal component. At the same time, reciprocity predisposes a development of mechanisms controlling interactions of reciprocal components themselves. From the spectrum (set) of an initial system’s variables, some of them split into components that increase the number of the system’s variables and, which is important, provide the broader spectrum of functional possibilities (outcomes) which initially was restricted by the smaller number of variables. In this process, other functions may undergo repression and eventually be eliminated from the genome.

A demonstrative example of bypassing long chains of metabolic reactions, which are “eliminated” during phylogenetic development, is a formation of substrate-enzyme complexes (SE) in order to obtain final products from the available substrates. Until the process of transformations is not activated by the surrounding environment and enzymes, each of the components of biochemical reaction is in an inertial state. It makes biochemical chains of reactions to be controllable and dependable of simultaneous availability of all required components. Because of the affinity, substrates form temporary complexes with enzymes alleviating distribution of biochemical energy in order to transform substrates into the products. After this process energetically and chemically is accomplished, a product of the reaction as well as participating in the reaction enzyme acquires stable states. Thus, enzymes functionally serve as intermediate agents (mediators) between substrates and products playing the role of the systems transforming input into output. Biochemical mechanisms of these processes are extremely complex and are involved in the phylogenetic development of the functional structure of BS in parallel with functional splitting.

It is postulated that any system (subsystem) has its reciprocal counterpart. This principle underlies mechanisms of encoding of a system’s functions in the genome during phylogenesis and decoding them in ontogenesis (individual development). Functional splitting should be understood as obtaining one-dimensional orthogonal characters (variables) that relate to each other reciprocally. The model of these relations is provided by “reciprocal” operator $$ {S}_1=\left(\begin{array}{cc}\hfill 1\hfill & \hfill \hfill \\ {}\hfill \hfill & \hfill -1\hfill \end{array}\right) $$.

The role of NFB in the splitting mechanism is mostly coordinating and signalling its accomplishment. The criterion of the accomplishment is a *closed cycle*. When a newly formed element is involved in metabolism as a part of the chain of biochemical reactions or it is an element of functional transformations in a more common sense, it fits the functional structure of a system if only its functional contribution is accepted, i.e. its involvement will allow the system to fulfil a newly formed functional circle that is warranted by closed NFB loops. Otherwise, the process will be presented as spiral, untwisting trajectories indicating that it is still unaccomplished and formally never will be accomplished because of the character of trajectories demonstrating growing unsteadiness (Fig. 5c).

Another aspect indicating the coordinating role of NFB in phylogenesis is its incapability to manage separately variables involved in NFB regulations. This is because NFB does not have one-dimensional invariant subspaces over the real numbers. It operates on elements as pairs of functions. From the physiological point of view, if in some conditions the value of one of the variables is changing, it will be accompanied by simultaneous changes of the states of another variable. NFB does not have differentiating features.

PFB as it was stressed above plays a “supplementary” role, because its matrix in the standard basis shows how a one-dimensional invariant subspace may contain both initial variables. Physiologically, it makes sense, because the matrix of PFB demonstrates capabilities of the regulatory structure to manage complex systems (tissues, organs, etc.) from simpler ones (biologic cells, biochemical molecules).

## Ontogenesis as a process of unfolding of the structure of a functional system

Ontogenesis is a stepwise process of involving differentiated components in the functional structure of BS during maturation. It can be partitioned according to the stages of maturation. When the system is transforming from one of the premature stages to the next level, new functional properties including metabolic pathways are being involved in the global functional structure. What makes this process complex is the necessity to reorganize the existing structure in order that the newly involved elements become functionally compatible with the existing ones. Although the system should be ready for the forthcoming changes, it is still a steady structure, meaning all three functional components have normal “functional images”, which in matrix language means all three matrices {*S*_0_, *S*_1_, *S*_2_} representing a regulatory structure are traceless and no “pathological changes” (*TrS*_*i*_ > 0, *detS*_*i*_ > 0) are affecting the matrix structure. When a system switches over to the other more complex level, it occurs by asymmetric division of stem cells (SC) as a leading mechanism. If, for example, only one character splits during differentiation, transformation to the next level will increase the dimensionality of the space of biologic variables (dimensionality of the module) in two units, for example, from *R* ^2^ to *R* ^4^ because of a pair of reciprocal functions, so that *m* steps will result in 2*m*-dimensional space. The set of matrices corresponding to the *n*-dimensional space of variables *L* ^*n*^ will be presented by *n* × *n* matrices belonging to *sl*(*n*, *R*), (*n*—even number).

A clue in the understanding of the mechanisms of cell differentiation lies in the *geometry of the space of transformations* of a normal system’s states (or in the structure of *Λ*module), which in general can be characterized as a three-dimensional space containing hyperbolic and symplectic subspaces. Phase trajectories of *n*-dimensional “diagonal” systems will correspond to an *l*-dimensional saddle with equal numbers of positive and negative eigenvalues and one-dimensional spheres, the direct sum of which will represent *k*-dimensional torus; *l* + *k* = *n*. The geometrical surface corresponding to the quadratic form of the elements of the *Λ*module is a three-dimensional hyperboloid, where each piece is represented by *n*-dimensional hyperbolic (for ${S}_1^{(n)}$ and ${S}_2^{(n)}$ matrices) and *n*-dimensional symplectic (for ${S}_0^{(n)}$ matrix) subspaces. The three-dimensional space of 2 × 2 matrices from *sl*(2, *R*) is equipped with an indefinite metric with signature (−, +, +). The space of split quaternions is more complicated because of its metric signature, which is as well indefinite (−, −, +, +). Subspaces of four-dimensional space of coquaternions may represent different metric properties, which can be positive definite and indefinite depending on combinations of composing components.

To sketch a mechanism of cell differentiation consider an *L* = *R* ^2^ module or a space of two biologic variables, the function of which is determined by a pool of cells which have not yet reached maturation. A functional module (space) *Λ* responsible for the functional structure of these cells is represented by 2 × 2 matrices $$ S=\left(\begin{array}{cc}\hfill a\hfill & \hfill c\hfill \\ {}\hfill b\hfill & \hfill d\hfill \end{array}\right),a+d=0 $$. Asymmetric division of stem cells and cell renewal processes add new functional features to the system, thus increasing dimension of the new space of variables *L*′ (from *R* ^2^ to *R* ^4^). The space of transformations *Λ*′ now is represented by 4 × 4 matrices $$ {S}^{(4)}=\left(\begin{array}{cc}\hfill A\hfill & \hfill \circ \hfill \\ {}\hfill \circ \hfill & \hfill -A\hfill \end{array}\right) $$, where the skew block-diagonal elements ∘ are some entries, not obviously zeroes.

As a block matrix consisting of 2 × 2 blocks, *S* ^(4)^ is a traceless matrix like *S*, but entries inside blocks, the total number of which is 16, indicate a more complex structure of *S* ^(4)^ compared to *S*. This is due to increased number of variables that dramatically increases the amount of functional components linking them.

In order to preserve morphological steadiness and functional stability, a maturer system (a system after new components are being involved) undergoes structural adaptation. It does not necessarily imply substantial qualitative changes in the structure but rather adjustment of the existing links. In matrix language, it means adjustment of the values of matrix entries in order to escape pathological regulatory patterns appearing when *TrS* ^(4)^ > 0 and *detS* ^(4)^ > 0.

It should be noticed that NFB regulatory loops have to be closed, i.e. phase trajectories should comprise closed circles. It is difficult to see it in representing a matrix *S* ^(4)^ structure until it is not in a diagonal form. If it contains positive values of diagonal entries linked by skew symmetric elements located on the secondary diagonal, the corresponding subsystem will behave according to an unsteady focus that will indicate its pathological development (Fig. 5c).

It is hypothesized that in cases of pathological development one of the chromosomal loci responsible for reciprocal relations is substituted by another one, so that corresponding to this locus, protein (or other biochemical structure) synthesized in the progeny cells will be involved in the metabolism in a way that instead of inhibiting the function of its reciprocal counterpart, will enhance or, most probably, not have an impact on it. Lack of the inhibiting actions will result in attempts (through NFB) to produce a normal biochemical substance, which is not possible on the considered level of gene expression. A synthesized malfunctioning protein will again turn on a chain of pathological reactions for which the involved reciprocal pair is responsible. Thus, the developed vicious circle will result in (uncontrollable) cell proliferation. It also will indicate on the functional instability of the system.

Consider a step of differentiation in which some subsystem is involved with a defective gene responsible for RL. In matrix form, after the matrix is transformed to the diagonal view by similarity transformations, the corresponding subsystem will have the view of a 2 × 2 matrix with the positive entries on the main diagonal and skew symmetric form on the secondary diagonal (Fig. 5c). This is because of the complex roots with the nonzero real parts obtained from the characteristic equation. The phase portrait of the system will demonstrate unsteady behaviour. The phase curves are an unsteady focus. It is easy to see that the matrix corresponding to the considered subsystem (after it is being normalized) can be decomposed on diagonal and skew diagonal forms $$ 1=\left(\begin{array}{cc}\hfill 1\hfill & \hfill \hfill \\ {}\hfill \hfill & \hfill 1\hfill \end{array}\right)+{S}_0=\left(\begin{array}{cc}\hfill \hfill & \hfill 1\hfill \\ {}\hfill -1\hfill & \hfill \hfill \end{array}\right) $$. A much simpler unsteady structure is obtained in the case of a diagonal matrix with different positive eigenvalues, which do not have negative reciprocal counterparts $$ S=\left(\begin{array}{cc}\hfill n\hfill & \hfill \hfill \\ {}\hfill \hfill & \hfill m\hfill \end{array}\right),n\ne m,n>0,m>0 $$, (Fig. 5b). In cases like that, phase trajectories correspond to an unsteady node. It is easy to see that despite adding a skew symmetric matrix (NFB) to the diagonal form with positive eigenvalues, the process remains unsteady: trajectories are not closed circles, but instead, they are displaced outward from the initial point showing untwisting spiral-shaped curves (Fig. 5c).

Cell proliferation may reflect a system’s response to various, including pathological, conditions. As it was mentioned before, it may manifest reparation as a stage of inflammatory reactions on microbial invasion. Cell proliferation is also a prominent feature of benign tumours. If cell proliferation is totally uncontrollable, it indicates on functional states, for which cancer growth is mostly responsible.

Cell differentiation has two interrelated components: cell proliferation as the system’s response on the process of maturation and cell integration (organized structure of tissues and organs as well as functionally capable organisms). A lack of cell integration is a key factor of cancer development.

## Alterations in the functional structure of biologic systems that predisposes cancer development

The biologic system is an hierarchical structure, and the ontogenetic process of an organism’s maturation follows hierarchical principles inherited by BS. Two general types of cell division, asymmetric and symmetric, describe vertical and horizontal directions in functional maturation, as well as indicate directions of the applied regulatory functions and points of their integration.

**Fig. 8:**
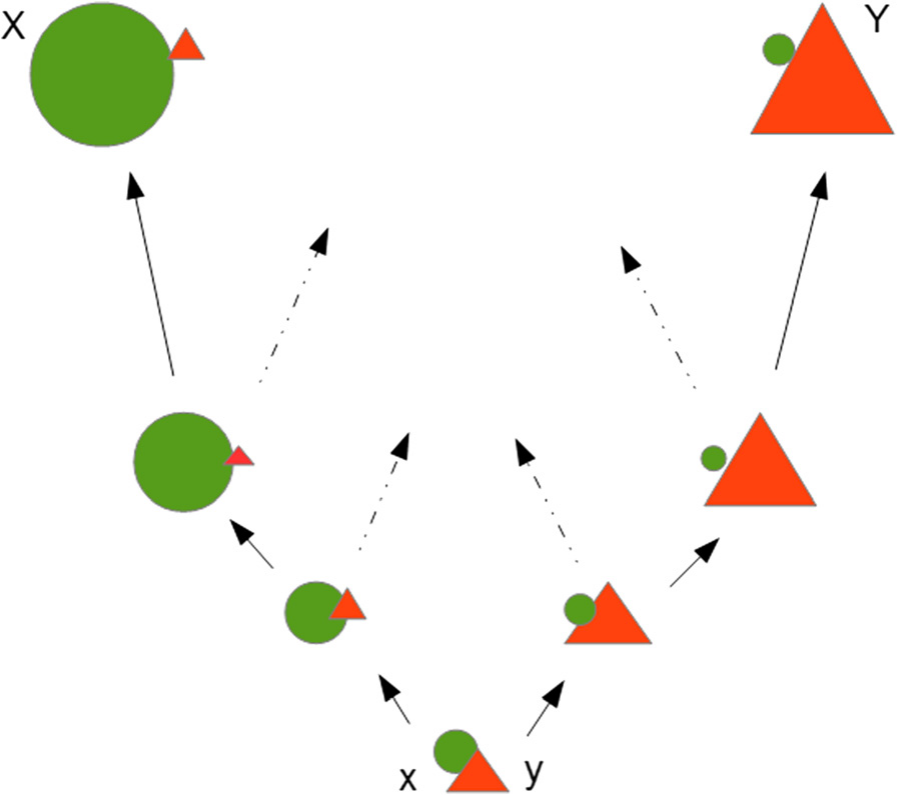
Schematic representation of phylogenetical splitting of relatively homogeneously distributed characters *x* and *y* (*green circle* and *red triangle*, respectively) at the bottom and clearly distinguishable functional components *X* and *Y* at the top, despite the presence of the initial, becoming rudimentary, counterparts (*small red triangle* and *green circle*)

Asymmetric division splits SC function, moving differentiated parts to the next level of the hierarchical scale. It splits the stem of characters on two reciprocally related branches, containing counterparts to each other originated from a progenitor character that underwent splitting (Fig. 8). Within each branch obtained as a result of functional splitting, a mechanism regulating apoptosis also exists. It is based on RL between a pool of renewed cells and a pool of old, malfunctioning, cells which enter the process of elimination. A capacity of a pool of renewed cells depends on functional demands, but velocity of cell elimination is determined by the functional conditions of cells themselves, i.e. an ability to obtain a certain amount of output. Lack of functionality of all inner components, indicating that cells are incapable of producing the required outcome (old cells), signals apoptotic mechanisms to start destroying malfunctioning cells. For example, when some components of cell function are preserved, it prevents active apoptosis, which is observed in cases of thyroid hyperplasia in both hyper- and hypothyroid conditions, when cell proliferation prevails over cell destruction. Partial deteriorations of thyrocytes that resulted in malfunctioning are not enough to trigger active apoptosis and bring the thyroid gland to its normal size.

Each type of biologic cells is unique in terms of molecular organization and functions, and these specific features allow them to establish functional links with other cells to form tissues and organs. Thus, these links organized in the functional structure of BS reflect mechanisms of functional and morphological integration of elements originated from different stems. Tissues and organs are examples of functional and morphological integration comprising several levels. Even a biologic cell itself is an integrative unit representing different levels of organization. Each mature cell contains information regarding links with elements of different hierarchical levels, and genes expressed in order to activate these links, in fact, trigger a functional structure of some hierarchical level where RL, NFB and PFB are the basis (and basic) components. An integration of basis components regulating metabolic flows reflects the integration of molecules, organelles in cells, cells in tissues and organs in organisms. Within each hierarchical layer, all three basis elements comprise a whole regulatory structure providing functional links among morphological elements. Each hierarchical level corresponds to some stage of differentiation. Structures located at the top belong to the current stage.

**Fig. 9:**
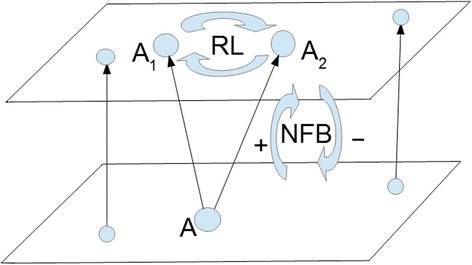
Two hierarchical levels of stem cell differentiation. Asymmetric cell division is resulted in splitting of the character *A* on two daughter characters *A*_1_ and *A*_2_ linked by reciprocal interactions (*RL*). Other characters may remain unchanged. Different stages of cell maturation are linked by negative feedback (*NFB*) sending inhibitory signals to the pool of cell progenitors

Links between different functional levels are provided by NFB mechanisms (Fig. 9). Each layer can also be considered as a level of functional acceleration of the previous level. It is supported by the nature of NFB, and related dynamic images obtained in the assumption that one of the variables is a variable itself, whereas another is its velocity [19]. It is also supported by thermodynamical laws, because in complex, multihierarchical biologic systems in order to conserve negative entropy metabolic machinery should possess metabolism-accelerating mechanisms. An example of that is a functional activating mechanism of the hypothalamic pituitary adrenal axis [24].

Symmetric cell division is resulted in the proliferation of cells of the same kind. Thus, it increases a population of cells and a functional capacity of the system to counteract and at the same time collaborate with the surrounding environment. Environment should be understood in a broad sense as any input from surrounding subsystems as well as impact from the physical environment. Reciprocal interactions occur within the same (horizontal) hierarchical level, whereas NFB links this level to the progenitor structures, which include corresponding stem cells (Fig. 9). Negative feedback (NFB) is a two-directional regulatory process; it connects reciprocal components of the upper level to the cambial structure on the lower level via inhibitory signals. Reciprocal elements send inhibitory signals to the reciprocal sites of cambial cells via NFB, whereas cambial elements send activating signals to both reciprocal elements of the next level. In the next stage of cell maturation, a new level will be maturer than the previous one, and the previous level becomes relatively premature compared to the following generation of more differentiated cells. The scheme of NFB and RL regulations will be the same as on the previous level. What substance(s) plays the role of inhibiting and activating transformers is unclear. Also, it is unclear what mediates reciprocal interactions.

Different hierarchical levels are interrelated. A system will transform its functional structure to the next level, if the functions of a previous level were successfully accomplished. A functional accomplishment means NFB interactions linking different levels are not integrated with pathological functional patterns and are represented by closed loops.

Normally involved substances serve as transducers linking chains of metabolic reactions. If the loop is closed, it means that the output signal returns back in the form of the “recognized” input signalling that there is a closed chain of metabolic transactions. Because of the closed chain of transformations, the returned signal is directly mediated by the system’s own output.

If synthesizing substances from the previous level are efficiently involved in the metabolism on the new level, inhibitory signals sent from both components of RL to the previous level will interact in a way that mimics substrates, enzymes and product (SEP) interactions. The substantial difference is that functional interactions between reciprocal elements are always bidirectional.

A component of the functional system from which a new reciprocal pair is originated will serve as an intermediate structure linking reciprocal elements. To be functionally congruent to the reciprocal elements, the part of the system will undergo functional adaptation. It also works when a system, in which a new reciprocal pair is involved, will serve as an intermediate structure (environment) linking reciprocal components.

In a mature organism, a pool of finally differentiated cells coexists with the pool of stem cells in different stages of functional differentiation. The pool of stem cells is not homogeneous, because immature cells are in different stages of maturation. They have different potency depending on the position on the scale of maturation. The more potent the cells are, the longer the way of further functional transformations until they become mature, and these transformations are mediated by NFB signals sent from more differentiated levels. It seems that the triggering mechanism should be sensitive to the diminished amount of functional outcome coming from the pool of mature cells. The lower (deeper) the level of deteriorated cambial cells, the more hierarchical levels are involved to reach the mature level, and the more vulnerable are mechanisms of linking elements during the cell renewal process to maintain morphological and functional structure of complex systems like tissues and organs. In cancer cells, functional links among components comprising biological tissues are broken (or lost). For example, adenocarcinomas arising from glandular cells destroy endothelial tissue due to missing of functional links making glandular cells integrated to other elements of the tissue. Because some of the metabolic links with the normal systems are preserved, cancer cells develop using them simply as a source of energy. In acute leukaemia, blood as a tissue loses its functionality due to the deteriorated integrative links among formed blood elements. It makes a pool of pathological blast cells (leukemic cells) dominate in the functional and morphological features of the blood.

Pathological conditions characterizing cancer growth are based on the deterioration of chromosomal loci responsible for reciprocal links. Reciprocal links (RL) are always originated from the previous levels of cell differentiation and correspond to the pool of stem cells giving rise to the considered population through asymmetric cell divisions. Following assymetric divisions vertical and horizontal levels are involved in cells' interactions. The mechanisms of cells' interactions contain components responsible for cell integration resulting in the formation of tissues and organs. A lack in a reciprocal counterpart diminishes the inhibitory stimuli on the cambial layer; hence, it activates proliferative stimulation of stem cells on mature hierarchical levels via NFB. Functional demands of a higher level will cause uncontrollable proliferation of cells responsible for malfunction. Because the reciprocal locus of the chromosome is deteriorated, instead of normal cells, stem cells (SC) will produce cells with broken reciprocal links. Broken links eventually launch a vicious circle via NFB resulting in uncontrollable proliferation with the formation of the whole pool of defective cells. Because of the lack of reciprocity, integration of proliferating cells with other cells belonging to the corresponding tissue will form malfunctioning tissue with structureless morphology.

All three basis regulatory elements (RL, NFB and PFB) are involved in the functional structure of cancer cells. Only on the level(s) of cell differentiation from which cancer stem cells (CSC) [2] are originated are reciprocal links deteriorated, leading to the population of cells unable to integrate with functionally and morphologically supplementary elements. SC, which are progenitors to the CSC, are normal and the functional structure of progenitor cells are embedded in the cancer cell structure according to hierarchical principles of system development. This is the main reason why cancer cells have autonomy, shared metabolic pathways and are not entirely recognized by the immune system as a foreign structure.

## Conclusions

The presented hypothetical model of mechanisms of cancer development is based on geometrical properties of the space of split quaternions where the split complex part is presented by the basis elements of Lie algebra *sl*(2, *R*). A real part presented by ±unit matrix allows describing unsteady states of BS which can be considered as a prominent feature of a system undergoing pathological development. Matrix multiplication as one of the ways to compose new elements of the coquaternion (split quaternion) ring indicates that pathological conditions could be a result of consequent actions of basis regulatory elements of BS such as RL, NFB and PFB. Among different scenarios in the development of a number of pathological states, it is shown that deterioration of reciprocal links is of crucial importance in cancer cell development. In matrix language, it corresponds to the transformation of the “reciprocal” matrix *S*_1_ to the unit matrix 1(*E*). Phase trajectories corresponding to the unit matrix is an unsteady node. Superposition of the unit matrix *E* with the matrices corresponding to NFB (*S*_0_) and PFB (*S*_2_) indicating the system’s integrative properties does not change the behaviour of the system, which remains unsteady.

Functional splitting seems to be a leading cause determining the phylogenetic development of different species which is “condensed” and inverted in the much shorter process, ontogenesis. Based on geometrical properties of the space of split quaternions, it was assumed that during organism maturation a pool of cambial (stem) cells in different premature stages gives rise to the next generation of more mature cells, thus forming a hierarchical structure, where biological variables are linked by elements of the space of transformations spanned by three basis regulatory elements of BS: NFB, RL and PFB, corresponding to *S*_0_, *S*_1_ and *S*_2_ matrices. While RL and PFB can be transformed to one another and operate within a given hierarchical level, NFB links different hierarchical levels.

If the chromosomal loci of stem cell(s) responsible for reciprocal interactions are altered, it may lead to the chain of functional and/or metabolic interactions caused by a lack of inhibitory stimulation of cambial layers and lead to the formation of a vicious circle resulting in the uncontrollable proliferation of malfunctioning cells. Because a reciprocal structure underlies the mechanisms of asymmetric cell division and formation of the following hierarchical levels, defects in chromosomal loci responsible for reciprocal links will result in the incapability of a system to integrate defective structural elements with normal anatomic and functional parts. Because progenitors to the level from which CSC are originated are normal SC and the structure of these cells are somehow involved in the functional structure of cancer cells, some links with other systems are preserved and cancer cells are not entirely recognized by the immune system as a foreign structure. Thus, instead of being involved in the local (intertissue) integration, cancer cells will only be capable of using some of the metabolic pathways as a source of energy.

The theoretical aspects of cancer development in the context of this work are linked to the geometrical structure of the space of split quaternions and its subspaces with positive definite (and indefinite) metrics that may explain some pathological changes in a normal system’s behaviour.

Future practical investigations could be aimed towards finding reciprocal loci of chromosomes and their related functions, as well as functional changes due to the deterioration of reciprocal counterparts. Also, in the scope, it seems interesting to find physiological correlates of the functional integration of RL, NFB and PFB structures.

## Abbreviations

BS: biologic systems
RL: reciprocal links
NFB: negative feedback
PFB: positive feedback
SC: stem cells
CSC: cancer stem cells
DCD: dysplastic cell disorders
ODE: ordinary differential equations
CVS: cardiovascular system

## Footnotes

1 Bifurcation of a system means qualitative change in the system’s behaviour caused by changes in the system’s parameters values. The obtained system (unsteady node) is not topologically equivalent to the saddle, because the saddle cannot be smoothly deformed to the unsteady node.

2 Although linearization of some nonlinear system may give ambiguous results, almost all nonlinear systems have linear analogues, which are much easier to analyse and see prominent features hidden in complicated nonlinear structures.
